# Accounting for biases in survey-based estimates of population attributable fractions

**DOI:** 10.1186/s12963-019-0196-6

**Published:** 2019-12-12

**Authors:** Ryan Masters, Eric Reither

**Affiliations:** 1 Department of Sociology, Population Program and Health & Society Program, Institute of Behavioral Sciences, University of Colorado Population Center, Boulder, CO 80309 USA; 2 Department of Sociology, Social Work, and Anthropology, Utah State University, Logan, USA

**Keywords:** Attributable fractions, Selection bias, Confounding bias, Mortality

## Abstract

**Background:**

This paper discusses best practices for estimating fractions of mortality attributable to health exposures in survey data that are biased by observed confounders and unobserved endogenous selection. Extant research has shown that estimates of population attributable fractions (PAF) from the formula using the proportion of deceased that is exposed (PAF_pd_) can attend to confounders, whereas the formula using the proportion of the entire sample exposed (PAF_pe_) is biased by confounders. Research has not explored how PAF_pd_ and PAF_pe_ equations perform when both confounding and selection bias are present.

**Methods:**

We review equations for calculating PAF based on either the proportion of deceased (pd) or the proportion of the entire sample (pe) that receives the exposure. We explore how estimates from each equation are affected by confounding bias and selection bias using hypothetical data and real-world survey data from the National Health Interview Survey–Linked Mortality Files, 1987–2011. We examine the association between cigarette smoking and all-cause mortality risk in the US adult population as an example.

**Results:**

We show that both PAF_pd_ and PAF_pe_ calculate the true PAF in the presence of confounding bias if one uses the “weighted-sum” approach. We further show that both the PAF_pd_ and PAF_pe_ calculate biased PAFs in the presence of collider bias, but that the bias is more severe in the PAF_pd_ formula.

**Conclusion:**

We recommend that researchers use the PAF_pe_ formula with the weighted-sum approach when estimates of the exposure-outcome relationship are biased by endogenous selection.

## Background

This paper discusses best practices for estimating the fraction of mortality attributable to health exposures in survey-based data that are biased by both observed confounders and unobserved endogenous selection. Much extant work has reviewed errors in computing population attributable fractions (PAFs) in the presence of confounders [1–6], but little work has considered how different formulae for computing PAFs are affected by endogenous selection biases (e.g., collider bias).

Endogenous selection bias can affect estimates of statistical associations in many ways. Conditioning on a collider variable—that is, a variable caused by two other variables that are associated with the exposure and the outcome—can occur through statistical control, stratification of the sample into different groups, or the selection of participants into a study [7–11]. Introducing collider variables through any of these mechanisms can bias estimates of associations between exposure and outcome. In this study, we focus on *unobserved* endogenous selection—a problem that commonly occurs in health studies through the sampling process of recruiting study participants. Simply put, the likelihood of participation in a health study can be affected by both the exposure and outcome, which can bias estimates of the true association between them.

The most common PAF formulae are based on either the *p*roportion of *d*eceased (pd) in the sample that receives the exposure or the *p*roportion of the *e*ntire sample (pe) that receives the exposure [1]. The two main aims of this investigation are to examine the performance of these model-based methods for calculating PAF in the presence of (1) known and observable confounders of the exposure-mortality association and (2) collider bias. We focus on the association between cigarette smoking and all-cause mortality risk in the US adult population, which is confounded by other variables and also a likely contributor to unobserved endogenous selection bias in survey-based data of smoking and mortality risk [7].

## Methods

We use hypothetical data and real-world survey data to calculate PAF in the presence of confounding and unobserved endogenous selection. In all of our exercises, non-exposed cases are respondents who have never smoked cigarettes and exposed cases are respondents who are current or former smokers. The association of interest is how smoking affects all-cause mortality risk. For each exercise, we estimate the fraction of US mortality attributable to cigarette smoking using the PAF_pd_ formula:

1 $$ {\mathrm{PAF}}_{\mathrm{pd}}=\left(\mathrm{pd}\ast \left(\mathrm{RR}-1\right)\right)/\mathrm{RR} $$

where pd is the prevalence of a health exposure among the deceased cases and RR is the mortality risk ratio between the exposed and non-exposed subjects [12]. We also estimate this fraction using the PAF_pe_ formula:

2 $$ {\mathrm{PAF}}_{\mathrm{pe}}=\left(\mathrm{pe}\ast \left(\mathrm{RR}-1\right)\right)/\left(1+\left(\mathrm{pe}\ast \left(\mathrm{RR}-1\right)\right)\right) $$

where pe is the prevalence of the exposure among all cases in the sample [6, 13]. For each formula, we adopt a “weighted-sum” approach [1, 3, 14, 15], which uses model-based adjusted estimators of PAF separately for each adjustment level *i* as well as the distribution of cases by the adjustment levels:

3 $$ {\mathrm{PAF}}_{\mathrm{pd}}=\sum {W}_i\ast \left({\mathrm{pd}}_i\ast \left({\mathrm{RR}}_i-1\right)\right)/{\mathrm{RR}}_i $$

4 $$ {\mathrm{PAF}}_{\mathrm{pe}}=\sum {W}_i\ast \left({\mathrm{pe}}_i\ast \left({\mathrm{RR}}_i-1\right)\right)/\left(1+{\mathrm{pe}}_i\ast \left({\mathrm{RR}}_i-1\right)\right) $$

where *i* indicates the adjustment level (i.e., confounder) and *W*_*i*_ indicates the proportion of deaths in adjustment level *i*. The weighted-sum approach is mathematically equivalent to the PAF_pd_ [1, 14]; combining the PAF_pd_ with the weighted-sum approach is therefore redundant. Nevertheless, we apply it in all of our exercises to maintain consistency.

### Exercise 1: Observed confounding bias in hypothetical data

In our first exercise, we examine PAF estimates from Eqs. (1–4) in the presence of a single confounder, race/ethnicity. For simplicity, we consider race/ethnicity using only two categories, non-Hispanic black and non-Hispanic white (hereafter black and white). The hypothetical data are composed of 1000 black respondents and 4000 white respondents. Both smoking prevalence (i.e., pe) and mortality risk are higher among black respondents than among white respondents, which confound the smoking-mortality association. In these data, black pe is 0.35 compared to white pe of 0.2, and overall mortality risk for black respondents is 0.3 compared to 0.2 for white respondents.

### Exercise 2: Unobserved endogenous selection bias in hypothetical data

Our second example uses the same data as before, but presupposes that estimates of the smoking-mortality association are biased by differential selection into the sample. We assume that current smokers sampled are relatively more select on health than are non-smokers. That is, both the non-smoking and the smoking samples are healthier than the true populations, but the difference between the smoking sample and the smoking population is greater than the difference between the non-smoking sample and the non-smoking population. This unobserved process of health selection biases downward the all-cause mortality RR estimated in the sample data. When these conditions hold, both PAF estimates will be biased due to the central role of the RRs (see Eqs. 1–4). Moreover, the distribution of deaths by exposure and by adjustment levels, *W*_*i*_, will also be biased. This is because counts of deaths among the exposure group in the sample will be artificially low and, consequently, *W*_*i*_ will be incorrect. Thus, PAF_pd_ and PAF_pe_ estimates will remain biased via *W*_*i*_ even if our adjusted RRs account for collider bias. Finally, the estimated PAF from the PAF_pd_ formula will be additionally biased, due to the central role of the pd in the calculation of the PAF. That is, the pd in the observed data, like the RRs in the observed sample data, will be downwardly biased because deaths among the smoking sample are underreported.

### Exercise 3: PAF estimation with real-world survey data

Finally, we illustrate the points above by analyzing the smoking-mortality association in the National Health Interview Survey–Linked Mortality Files (NHIS-LMF) for years 1987–2009. These data are composed of NHIS waves from 1987 and 1989–2009 that have been linked to official death records at the National Death Index through December 31, 2011 (the 1988 NHIS survey did not contain information about respondents’ smoking behavior). The NHIS-LMF are designed to form a representative sample of non-institutionalized US adults [12]. To simplify the example, we limit the analytic sample to contain only US adult black and white men and women aged 40 through 84 at time of interview and whose survival is followed between ages 50 and 84. We extend the example by considering two levels of smoking exposure, “former smoker” and “current smoker,” and by considering three possible confounders of the smoking-mortality association: race/ethnicity (i.e., white and black), gender (i.e., men and women), and age group (i.e., 50–59, 60–69, 70–79, and 80–84).

We fit a series of clog-log discrete-time survival models to estimate smoking-based differences in US adult mortality risk. First, we fit a *baseline* model that estimates differences in mortality risks between current, former, and never smokers (reference category). Next, we fit a *confounder* model that estimates age-specific differences in mortality risks between current, former, and never smokers, adjusting for race/ethnicity and gender as categorical confounders of the smoking-mortality association. We also fit models separately for black and white men and women that estimate age-specific RRs for former and current smokers compared to never smokers (i.e., confounder-specific models to be used with the weighted-sum approach to calculate PAFs). Finally, we fit a *bias* model that refits the *confounder* model by accounting for cohort-based variation in mortality risk and age-related selection biases in the NHIS-LMF data.

Participants in health surveys like the NHIS are positively selected on survival, health, and non-institutional living arrangements [16]. These selection biases tend to grow stronger with increasing age [17]. Thus, older respondents in NHIS-LMF data are selected on the outcome of interest (i.e., survival) and inclusion in the NHIS sampling frame (i.e., healthy and living in non-institutionalized housing). Combined, the selective nature of the sample results in collider biases via age-related selection into the sampling frame and the selective factors associated with age are likely stronger among respondents with health risk factors such as smoking than among healthy respondents [8].

Survival models fitted separately by cohort of entry into the NHIS sample provide evidence consistent with these assumptions about collider biases. For example, the estimated RR between current smokers and never smokers who died at age 70–80 ranges from 1.51 [1.43–1.58 95%CI] among respondents surveyed at age 70–75 to 4.11 [3.02–5.57 95%CI] among respondents surveyed at age 50–55. The *bias* model is a shared frailty survival model that estimates random effects variation in mortality risk by NHIS respondents’ 5-year age cohorts at the time of sampling. Overall, the model fits age-specific mortality risks separately for current, former, and never smokers, adjusting for gender, race/ethnicity, birth year, and random effects for a 5-year cohort of entry into the data.

Mortality differences between US adults self-reported to be current, former, and never smokers between ages 50 and 84 are estimated across these three models. We use the adjusted RRs between (1) current smokers and never smokers and (2) former smokers and never smokers, which are estimated from confounder-specific survival models and the weighted-sum approach to calculate the PAF for smoking as a cause of death in the US adult black and white populations between ages 50 and 84 for years 1987–2011. For all models, we contrast PAFs calculated from PAF_pd_ with PAFs calculated from PAF_pe_ to examine how each formula is affected by (1) confounders in the estimated smoking-mortality association and (2) collider bias.

The NHIS-LMF data analyzed for the current study are public-use files made available by the NCHS (https://www.cdc.gov/nchs/data-linkage/mortality.htm). The analytic scripts (Additional file 1) and calculations to generate results (Additional file 2) for Exercise 3 are available in the appendix.

## Results

### Exercise 1: Observed confounding in hypothetical data

The confounding effect of race/ethnicity on the smoking-mortality association is illustrated in Table 1. The all-cause mortality RR for smoking when unadjusted for the confounding effects of race/ethnicity is (450/1150)/(650/3850) = 2.32. Alternatively, the RR adjusted for race/ethnicity is 2.23. That is, when we estimate separate RRs for each race/ethnicity sample, we observe

**Table 1:** Hypothetical sample data

	Non-smoker	Smoker	Total	Counterfactual	pe	pd	RR
Non-Hispanic black							
Survived	500	200	700	769			
Died	150	150	300	231			
Total	650	350	1000	1000	0.35	0.50	1.86
*q*_*x*_	0.231	0.429	0.30				
Non-Hispanic white							
Survived	2700	500	3200	3375			
Died	500	300	800	625			
Total	3200	800	4000	4000	0.20	0.375	2.40
*q*_*x*_	0.156	0.375	0.20				
Combined							
Survived	3200	700	3900	4144			
Died	650	450	1100	856			
Total	3850	1150	5000	5000	0.23	0.409	2.32
*q*_*x*_	0.169	0.391	0.22				

*pe* proportion smoker in entire sample, *pd* proportion smoker among deceased, *q*_*x*_ probability of death, *RR* risk ratio

$$ \mathrm{Non}-\mathrm{Hispanic}\ \mathrm{black}=\left(150/350\right)/\left(150/650\right)=1.86 $$

$$ \mathrm{Non}-\mathrm{Hispanic}\ \mathrm{white}=\left(300/800\right)/\left(500/3200\right)=2.40 $$

When these race/ethnic-specific RRs for smoking are standardized by the race/ethnic distribution of deaths and the race/ethnic distribution of smoking prevalence, the adjusted RR is 2.23. If one does not account for the confounding effects of race/ethnicity on both mortality risk and the probability of smoking, one would incorrectly estimate the PAF by the following:

- Aggregating the probability of smoking to be (1150/5000) = 0.23,
- Aggregating the probability of smoking among decedents to be (450/1100) = 0.41, and
- Aggregating the RR associated with smoking to be (450/1150)/(650/3850) = 2.32.

As a result, estimates of the PAF for smoking, irrespective of the formula used, would be biased by not attending to the confounding effects of race/ethnicity:

$$ {\mathrm{PAF}}_{\mathrm{pd}}=\left(\mathrm{pd}\ast \left(\mathrm{RR}-1\right)\right)/\mathrm{RR}=\left(0.41\ast \left(2.32-1\right)\right)/2.32=0.233 $$

$$ {\mathrm{PAF}}_{\mathrm{pe}}=\left(\mathrm{pe}\ast \left(\mathrm{RR}-1\right)\right)/\left(1+\left(\mathrm{pe}\ast \left(\mathrm{RR}-1\right)\right)\right)=\left(0.23\ast \left(2.32-1\right)\right)/\left(1+\left(0.23\ast \left(2.32-1\right)\right)\right)=0.233 $$

The actual PAF shown in the counterfactual example above is (1100 − 856)/1100 = 0.222

Thus, by failing to account for (1) the higher prevalence of smoking among black respondents and (2) the higher mortality risks among black respondents, we would incorrectly inflate the RR associated with smoking and misattribute numerous deaths to smoking as a cause of mortality in the population. As such, it is necessary to identify the RR by accounting for confounders in model estimates, and then use this confounder-adjusted RR to calculate PAFs [18]. It has been argued that only the PAF_pd_ formula can accurately estimate the PAF when using confounder-adjusted RRs [3–6]. Yet, as others have noted, one can use the PAF_pe_ equation with the confounder-adjusted RR to derive the true PAF [1, 15]. To do so, one needs to first estimate separate PAFs for each confounder group (i.e., each adjustment level *i*), and then standardize these confounder-specific PAFs by the distribution of deaths across groups (i.e., *W*_*i*_).

To illustrate, when we estimate separate PAFs for black and white respondents, we see for black:

$$ {\mathrm{PAF}}_{\mathrm{pd}}=\left(\mathrm{pd}\ast \left(\mathrm{RR}-1\right)\right)/\mathrm{RR}=\left(0.5\ast \left(1.86-1\right)\right)/1.86=0.231 $$

$$ {\mathrm{PAF}}_{\mathrm{pe}}=\left(\mathrm{pe}\ast \left(\mathrm{RR}-1\right)\right)/\left(1+\left(\mathrm{pe}\ast \left(\mathrm{RR}-1\right)\right)\right)=\left(0.35\ast \left(1.86-1\right)\right)/\left(1+\left(0.35\ast \left(1.86-1\right)\right)\right)=0.231 $$

and for white:

$$ {\mathrm{PAF}}_{\mathrm{pd}}=\left(\mathrm{pd}\ast \left(\mathrm{RR}-1\right)\right)/\mathrm{RR}=\left(0.375\ast \left(2.40-1\right)\right)/2.40=0.219 $$

$$ {\mathrm{PAF}}_{\mathrm{pe}}=\left(\mathrm{pe}\ast \left(\mathrm{RR}-1\right)\right)/\left(1+\left(\mathrm{pe}\ast \left(\mathrm{RR}-1\right)\right)\right)=\left(0.2\ast \left(2.40-1\right)\right)/\left(1+\left(0.2\ast \left(2.40-1\right)\right)\right)=0.219 $$

To estimate the total PAF, we further attend to the distribution of deaths across groups. That is, we simply weight the confounder-specific PAFs by the proportion of total deaths occurring in the confounder groups (i.e., *W*_*i*_) [18]. The proportion of the total deaths that occurred among black respondents = (300/1100) = 0.273 and the proportion of total deaths that occurred among white respondents = (800/1100) = 0.727. When we weight the confounder-specific PAFs by the proportion of deaths in the two groups, *W*_*i*_, we retrieve the true overall PAF:

$$ {\mathrm{PAF}}_{\mathrm{NHB}}\ast {\mathrm{W}}_{\mathrm{NHB}}+{\mathrm{PAF}}_{\mathrm{NHW}}\ast {\mathrm{W}}_{\mathrm{NHW}}=\left(0.231\ast 0.273\right)+\left(0.219\ast 0.727\right)=0.222 $$

This shows that the weighted-sum approach can calculate the true PAF regardless if one uses the PAF_pd_ or PAF_pe_ formula. So long as (1) unobservable confounders or unobservable selection do not induce bias, and (2) one attends to observable confounders of the smoking-mortality association, one can use adjusted RRs with either PAF_pd_ or PAF_pe_ and the weighted-sum approach to calculate the PAF for smoking-related mortality in the sample [18].

### Exercise 2: Unobserved endogenous selection in hypothetical data

In the next exercise, we extend the previous example to consider sample data that are biased by unobserved selection, causing underestimation of mortality risk in the smoking population. To simplify matters, let us assume that the prevalence of smoking is the same in both the sample and population so that the only change pertains to *q*_*x*_ for smokers in the sample. The new information about population parameters is presented in Table 2 below.

**Table 2:** Hypothetical population data

	pe	pd	RR	*q*_*x*_ (NS)	*q*_*x*_ (S)	*q*_*x*_ (total)
Non-Hispanic black	0.35	0.538	2.17	0.231	0.50	0.325
Non-Hispanic white	0.20	0.444	3.21	0.156	0.50	0.225

*pe* proportion smoker in population, *pd* proportion smoker among deceased in population, *q*_*x*_ *(NS)* probability of death among nonsmokers in population, *q*_*x*_ *(S)* probability of death among smokers in population, *RR* risk ratio

The mortality probabilities for the non-smoking populations equal those in the sample data (0.231 among blacks and 0.156 among whites). Smoking prevalence is also the same (pe_NHB_ = 0.35 and pe_NHW_ = 0.20). However, we now see discrepancies in the mortality risks for the smoking populations (0.500 in the white population vs. 0.375 in the white sample, and 0.500 in the black population vs. 0.429 in the black sample). These, in turn, affect the RRs for smoking (e.g., 2.17 vs. 1.86 for black and 3.21 vs. 2.40 for white), the pds (0.538 vs. 0.500 for black and 0.444 vs. 0.375 for white), and the *W*_*i*_ (e.g., 0.265 of population deaths are among blacks vs. 0.273 of sample deaths).

The confounder-specific PAFs using both the PAF_pd_ and PAF_pe_ formulae are as follows (estimates might be slightly different due to rounding):

non-Hispanic black:

$$ {\mathrm{PAF}}_{\mathrm{pd}}=\left(\mathrm{pd}\ast \left(\mathrm{RR}-1\right)\right)/\mathrm{RR}=\left(0.538\ast \left(2.17-1\right)\right)/2.17=0.290 $$

$$ {\mathrm{PAF}}_{\mathrm{pe}}=\left(\mathrm{pe}\ast \left(\mathrm{RR}-1\right)\right)/\left(1+\left(\mathrm{pe}\ast \left(\mathrm{RR}-1\right)\right)\right)=\left(0.35\ast \left(2.17-1\right)\right)/\left(1+\left(0.35\ast \left(2.17-1\right)\right)\right)=.290 $$

non-Hispanic white:

$$ {\mathrm{PAF}}_{\mathrm{pd}}=\left(\mathrm{pd}\ast \left(\mathrm{RR}-1\right)\right)/\mathrm{RR}=\left(0.444\ast \left(3.21-1\right)\right)/3.21=0.306 $$

$$ {\mathrm{PAF}}_{\mathrm{pe}}=\left(\mathrm{pe}\ast \left(\mathrm{RR}-1\right)\right)/\left(1+\left(\mathrm{pe}\ast \left(\mathrm{RR}-1\right)\right)\right)=\left(0.20\ast \left(3.21-1\right)\right)/\left(1+\left(0.20\ast \left(3.21-1\right)\right)\right)=0.306 $$

Standardizing these confounder-specific PAFs by the distribution of deaths, *W*_*i*_, we use the weighted-sum approach to calculate the true PAF:

$$ \left(0.290\ast 0.2653\right)+\left(0.306\ast 0.7347\right)=0.301 $$

We see that the PAFs in the sample data underestimate the true PAF in the population (0.222 vs. 0.301), and this bias is the same in the PAF_pd_ and PAF_pe_ formulae. The discrepancy arises from one’s inattention to (unobservable) endogenous selection bias in the sample data, resulting in biased sample estimates of the mortality RRs associated with smoking as well as biased *W*_*i*_ in the sample.

Imagine that we had accounted for unobservable selection bias in our survival models and correctly identified the RRs for smoking for both the black and white samples. Even though the adjusted RRs would be correct in our survival models, the counts of deaths in the sample data would remain biased. Consequently, the pd values in the sample stay at 0.50 and 0.375, and the proportion of deaths occurring among blacks and whites stay at 0.273 and 0.727, respectively. As a result, if we were to calculate the PAF using the adjusted RRs with PAF_pd_, we would find

$$ {\mathrm{PAF}}_{\mathrm{pdb}}=\left(\mathrm{pd}\ast \left(\mathrm{RR}-1\right)\right)/\mathrm{RR}=\left(0.50\ast \left(2.17-1\right)\right)/2.17=0.270 $$

$$ {\mathrm{PAF}}_{\mathrm{pdw}}=\left(\mathrm{pd}\ast \left(\mathrm{RR}-1\right)\right)/\mathrm{RR}=\left(0.375\ast \left(3.21-1\right)\right)/3.21=0.258 $$

The confounder-specific PAFs are biased (i.e., 0.270 estimated vs. 0.290 actual for blacks and 0.258 estimated vs. 0.306 actual for whites) even when using the adjusted RRs. Furthermore, when we use the weighted-sum approach and standardize these PAFs by *W*_*i*_, we add another source of bias because the distribution of deaths in each confounder group is biased as well: total PAF = (0.270*0.273) + (0.258*0.727) = 0.263. Yet, were we to follow conventional wisdom [3–6] and use the adjusted RR with the PAF_pd_ for the entire sample, we would estimate the same biased PAF:

$$ {\mathrm{PAF}}_{\mathrm{pd}}=\left(\mathrm{pd}\ast \left(\mathrm{RR}-1\right)\right)/\mathrm{RR}=\left(\left(450/1100\right)\ast \left(2.8-1\right)\right)/2.8=0.263 $$

Thus, even if we accurately accounted for selection bias in our survival models and estimated an unbiased RR (e.g., by fitting frailty models that account for selection bias in the smoking RR [19]), the PAF calculated from the PAF_pd_ formula will still be biased. In this case, a biased 0.263 is estimated for the sample when the true PAF in the population is 0.301 (a bias on the proportionate scale of 12.6%: (0.263 − 0.301)/0.301).

If we calculate the PAF using the confounder- and selection-adjusted RRs with the PAF_pe_ formula, we find

$$ {\mathrm{PAF}}_{\mathrm{peb}}=\left(\mathrm{pe}\ast \left(\mathrm{RR}-1\right)\right)/\left(1+\left(\mathrm{pe}\ast \left(\mathrm{RR}-1\right)\right)\right)=\left(0.35\ast \left(2.17-1\right)\right)/\left(1+\left(0.35\ast \left(2.17-1\right)\right)\right)=0.290 $$

$$ {\mathrm{PAF}}_{\mathrm{pew}}=\left(\mathrm{pe}\ast \left(\mathrm{RR}-1\right)\right)/\left(1+\left(\mathrm{pe}\ast \left(\mathrm{RR}-1\right)\right)\right)=\left(0.2\ast \left(3.21-1\right)\right)/\left(1+\left(0.2\ast \left(3.21-1\right)\right)\right)=0.306 $$

We see that the confounder-specific PAFs are *unbiased*. Only when we standardize these PAFs by the distribution of deaths, *W*_*i*_, do we introduce slight bias in the total PAF = (0.290*0.273) + (0.306*0.727) = 0.302 (a bias on the proportionate scale of − 0.3%: (0.301 − 0.302)/0.302). Thus, when we account for selection bias in our survival models and estimate unbiased adjusted RRs, the PAF calculated from PAF_pe_ will be biased, but only via *W*_*i*_. By using the PAF_pe_ equation, we avoid bias in estimates from the pd and dramatically reduce the overall bias in the PAF estimate (0.3% vs. 12.6%).

To recap, when sample data are biased by unobserved selection, both the PAF_pd_ formula and the PAF_pe_ formula will calculate a biased PAF—even if researchers adjust for selection bias in the data. However, the PAF_pd_ formula is far more affected by the bias than is the PAF_pe_ formula because bias is introduced in both the pd and *W*_*i*_. Conversely, estimates of the confounder-specific PAF from the PAF_pe_ equation are not biased, but some bias is introduced in the weighted-sum approach via *W*_*i*_. Theoretically, one could completely eliminate bias by identifying the true RR (i.e., attend to both observable confounders and unobservable selection biases) and standardizing the PAFs by the true distribution of deaths for each adjustment level (i.e., use population data to estimate *W*_*i*_).

### Exercise 3: PAF estimation with real-world survey data

For the final exercise, we calculate PAF for smoking as a cause of US adult mortality in the NHIS-LMF data, which are biased by confounding (i.e., age, race/ethnicity, and gender) and likely biased by endogenous selection (i.e., likelihood of sample inclusion depends on health). Table 3 shows age-specific mortality risks between years 1987 and 2011 for NHIS respondents who are current, former, and never smokers. The pd for former smokers (0.352) combined with the pd for current smokers (0.338) indicates that nearly 70% of the deceased NHIS sample had been exposed to smoking.

**Table 3:** Age-specific mortality counts by smoking exposure level, NHIS-LMF 1987–2009

Age	Dead	Total	*q*_*x*_	pe	pd
Never smokers					
50	1169	43,737	0.027	0.403	0.243
60	2109	41,443	0.051	0.396	0.233
70	4833	26,249	0.184	0.401	0.299
80	4705	10,030	0.469	0.451	0.414
Total	12,816	121,459	0.106	0.403	0.310
Former smokers					
50	959	27,283	0.035	0.251	0.200
60	2624	32,554	0.081	0.311	0.289
70	6310	24,207	0.261	0.369	0.391
80	4673	8649	0.540	0.389	0.412
Total	14,566	92,693	0.157	0.308	0.352
Current smokers					
50	2674	37,632	0.071	0.346	0.557
60	4335	30,727	0.141	0.293	0.478
70	4998	15,067	0.332	0.230	0.310
80	1977	3543	0.558	0.159	0.174
Total	13,984	86,969	0.161	0.289	0.338

From the sample data in Table 3, we calculate the unadjusted RRs:

$$ \mathrm{Total}\ {\mathrm{RR}}_{\mathrm{former}}=\left(14,566/92,693\right)/\left(12,816/\mathrm{121,459}\right)=1.489 $$

$$ \mathrm{Total}\ {\mathrm{RR}}_{\mathrm{current}}=\left(13,984/86,969\right)/\left(12,816/\mathrm{121,459}\right)=1.524 $$

Because we are calculating a PAF for two-levels of an exposure, former smokers and current smokers, the PAF formulae change slightly [18, 20]:

$$ {\mathrm{PAF}}_{\mathrm{pd}}={\mathrm{pd}}_{\mathrm{former}}\ast \left({\mathrm{RR}}_{\mathrm{former}}-1\right)/{\mathrm{RR}}_{\mathrm{former}}+{\mathrm{pd}}_{\mathrm{current}}\ast \left({\mathrm{RR}}_{\mathrm{current}}-1\right)/{\mathrm{RR}}_{\mathrm{current}} $$

$$ {\mathrm{PAF}}_{\mathrm{pe}}=\left({\mathrm{pe}}_{\mathrm{former}}\ast \left({\mathrm{RR}}_{\mathrm{former}}-1\right)+{\mathrm{pe}}_{\mathrm{current}}\ast \left({\mathrm{RR}}_{\mathrm{current}}-1\right)\right)/\left(1+\left({\mathrm{pe}}_{\mathrm{former}}\ast \left({\mathrm{RR}}_{\mathrm{former}}-1\right)+{\mathrm{pe}}_{\mathrm{current}}\ast \left({\mathrm{RR}}_{\mathrm{current}}-1\right)\right)\right) $$

$$ {\mathrm{PAF}}_{\mathrm{pd}}=0.352\ast \left(1.489-1\right)/1.48+0.338\ast \left(1.524-1\right)/1.52=0.232 $$

$$ {\mathrm{PAF}}_{\mathrm{pe}}=\left(0.308\ast \left(1.489-1\right)+0.289\ast \left(1.524-1\right)\right)/\left(1+\left(0.308\ast \left(1.489-1\right)+0.289\ast \left(1.524-1\right)\right)\right)=0.232 $$

We see that if we did not consider age, race/ethnicity, or gender as confounders of the smoking-mortality association in these NHIS-LMF data, we would estimate about 23% of US black and white adult deaths between ages 50 and 85 for years 1987–2011 were attributable to cigarette smoking.

Average RRs for current smoking estimated from clog-log discrete time hazard models are presented in Table 4, and overall PAFs estimated from the PAF_pe_ and PAF_pd_ formula are included as well.

**Table 4:** Estimated age-specific mortality risk ratios for current smokers relative to never smokers, NHIS-LMF 1987–2009

Age	Baseline model	Confounder model	Bias model
	RR	RR	RR
50–59	1.52	2.62	2.80
60–69	1.52	2.69	3.22
70–79	1.52	1.77	2.75
80–84	1.52	1.18	1.89
PAF_pe_	0.232	0.247	0.326
PAF_pd_	0.232	0.247	0.289

Note: RR abbreviation for risk ratio; RRs for the confounder model and bias model are standardized and averaged from survival models fitted separately to non-Hispanic black and white men and women

The *baseline* model estimates mortality risks for former and current smokers relative to never smokers that match the RRs observed in Table 3 (i.e., 1.49 and 1.52, respectively). Using these RRs, we estimate the same 0.232 PAF for smoking as a cause of US adult mortality, regardless if we estimate the PAF from the PAF_pe_ formula or the PAF_pd_ formula. The *confounder* model estimates age-specific RRs for former and current smokers relative to never smokers while controlling for confounding by gender and race/ethnicity. The age patterns in the RRs for current smokers suggest that the mortality consequences of smoking significantly decline with age. For example, current smokers are estimated to have about 2.6 to 2.7 times the mortality risk as never smokers in age-groups 50–59 and 60–69, but only about 1.2 times the mortality risk in age-group 80–84. When using these confounder-adjusted and age-specific RRs for smoking, we estimate a 0.247 PAF for smoking as a cause of US adult mortality.

Finally, the estimated age-specific RRs from the *bias* model are significantly larger than the age-specific RRs from the *confounder* model, especially at older ages. Although the smoking-mortality relationship attenuates with age, it is substantially less than the attenuation observed in the *confounder* model. Using these confounder- and selection-adjusted RRs, we calculate a PAF of 0.289 from the PAF_pd_ formula and a PAF of 0.326 from the PAF_pe_ formula. This is the only case in which we observe different PAF values depending on the formula used. This is because the PAF_pd_ formula remains biased by pd and likely underestimates the amount of mortality attributable to cigarette smoking in the US adult population. In this case, the PAF estimated from the PAF_pd_ formula is likely additionally biased by − 11.3% over the PAF_pe_ (0.289 − 0.326)/0.326) because it does not fully account for collider bias in estimates of the smoking-mortality association in the NHIS-LMF data.

## Discussion

Between-group differences in mortality (e.g., smokers and non-smokers) estimated from survey data are often biased by unobserved endogenous selection [8, 10]. These biases can distort research findings and lead to incorrect conclusions and misguided policy recommendations. Researchers should therefore be wary of collider biases and, when possible, adjust estimates to account for them. Relatedly, researchers should be wary of how these biases affect PAF calculations. In this paper, we demonstrated that the PAF_pd_ formula is far more sensitive to collider bias than the PAF_pe_ formula. Results from both our hypothetical examples and real-world illustration using the NHIS-LMF show the PAF_pd_ formula calculated severely biased estimates of the PAF for smoking as a cause of mortality. As such, if estimates of the exposure-outcome association are likely biased by endogenous selection, researchers should consider calculating PAFs using the PAF_pe_ formula with the weighted-sum approach. The main challenge to using the weighted-sum approach is the data required to scale estimates by *W*_*i*_, which increase with the number of confounders in the model. In addition, the weighted-sum approach may not be appropriate in small samples because estimates of *W*_*i*_ are unreliable [1].

The findings are important for researchers aiming to estimate the mortality burden of exposures that may induce collider bias in sample data. For example, estimates from the NHIS-LMF data indicate that widening educational disparities in US adult mortality have greatly increased deaths attributable to low educational attainment [21]. Yet, estimates of the education-mortality association in the NHIS-LMF data may be biased by mortality and health selection across age [22]. Deaths attributable to low education in the USA may, in fact, be underestimated by not accounting for collider bias in PAF calculations. Also, researchers have reported discrepant PAFs for obesity as a cause of US mortality. For example, Flegal et al. [5] review PAF values indicating 2–15% of adult deaths are attributable to high BMI. The discrepancies likely reflect the extent to which researchers attend to confounder and collider biases in model estimates and how these biases affect PAF calculations. While Flegal et al. ([5] p. 203) consider the PAF_pe_ to be “the invalid formula” and PAF_pd_ to be the “formula appropriate for use with adjusted relative risks when confounding exists,” their review did not consider how the PAF formulae were affected by collider bias. Results here indicate that the PAF_pd_ is, in fact, the formula that calculates more biased estimates when relative risks are adjusted for confounding and selection biases.

## Conclusion

Many studies have addressed best practices for calculating and interpreting PAFs for causes of mortality [1, 3, 5, 6, 20, 23–25]. In this paper, we extend these discussions to consider how unobserved endogenous selection bias (e.g., collider bias) distorts calculations of PAFs in the PAF_pd_ and PAF_pe_ formulae. Prior research has highlighted the importance of confounding bias in PAF calculations, but it has not considered how collider bias may affect PAF calculations. We used both hypothetical and real-world data on the smoking-mortality relationship to explore these considerations. Results from our examples demonstrate that both the PAF_pd_ and PAF_pe_ formulae can equally attend to observable confounders and accurately calculate PAFs via the weighted-sum approach [1, 3, 18]. Yet, the PAF_pe_ formula via the weighted-sum approach is preferred to the PAF_pd_ formula if RR estimates for the exposure are biased from endogenous selection. In contrast to conventional wisdom that recommends using the PAF_pd_ formula with adjusted RRs [3, 5, 6], we conclude by recommending the use of the PAF_pe_ formula with the weighted-sum approach when using RRs adjusted for both confounding bias and selection bias.

## Supplementary information

**Additional file 1.** This is a Stata do-file containing commands to fit clog-log discrete time survival models.

**Additional file 2.** This is a Microsoft Excel file containing race/ethnic- and gender-specific PAF estimates used to estimate PAF_pe_ and PAF_pd_ in Table 4.

## Data Availability

The datasets supporting the conclusions of this article are available as public use files of the NHIS-LMF data (https://www.cdc.gov/nchs/data-linkage/mortality-public.htm). The analytic scripts are available as “Additional file 1” and the PAF calculations are made available as excel files as “Additional file 2.”

## Abbreviations

95%CI: 95% confidence interval
NHIS-LMF: National Health Interview Survey–Linked Mortality Files
PAF: Population attributable fraction
PAF_pd_: Population attributable fraction estimated from the formula using the proportion of deceased that is exposed
PAF_pe_: Population attributable fraction estimated from the formula using the proportion of sample that is exposed
pd: The proportion of deceased exposed
pe: The proportion of sample exposed
*q*_*x*_ (NS): Probability of death among nonsmokers in population
*q*_*x*_ (S): Probability of death among smokers in population
*q*_*x*_: Probability of death
RR: Risk ratio
*W*_*i*_: The proportion of total deaths in each adjustment level
